# Strong, weak and neuron type dependent lateral inhibition in the olfactory bulb

**DOI:** 10.1038/s41598-018-38151-9

**Published:** 2019-02-07

**Authors:** Ronit Shmuel, Lavi Secundo, Rafi Haddad

**Affiliations:** 1 Gonda Multidisciplinary Brain Research Center, Bar-Ilan University, Ramat-Gan, Israel; 2 Department of Neurobiology, Weizmann Institute of Science, Rehovot, 7610001 Israel

## Abstract

In many sensory systems, different sensory features are transmitted in parallel by several different types of output neurons. In the mouse olfactory bulb, there are only two output neuron types, the mitral and tufted cells (M/T), which receive similar odor inputs, but they are believed to transmit different odor characteristics. How these two neuron types deliver different odor information is unclear. Here, by combining electrophysiology and optogenetics, it is shown that distinct inhibitory networks modulate M/T cell responses differently. Overall strong lateral inhibition was scarce, with most neurons receiving lateral inhibition from a handful of unorganized surrounding glomeruli (~5% on average). However, there was a considerable variability between different neuron types in the strength and frequency of lateral inhibition. Strong lateral inhibition was mostly found in neurons locked to the first half of the respiration cycle. In contrast, weak inhibition arriving from many surrounding glomeruli was relatively more common in neurons locked to the late phase of the respiration cycle. Proximal neurons could receive different levels of inhibition. These results suggest that there is considerable diversity in the way M/T cells process odors so that even neurons that receive the same odor input transmit different odor information to the cortex.

## Introduction

In many sensory systems, sensory information is processed locally and then transmitted to higher cortical regions in parallel by different cell types. In the mammalian olfactory bulb (OB) the two projection neurons, the mitral and tufted cells (M/T cells), are also believed to form two parallel streams of information. This notion has been supported by recent findings showing that mitral and tufted cells differ in some of their odor response properties and project to distinct cortical regions^1–5^. However, it is still unclear how neurons that share similar inputs vary in their output information, and which mechanisms govern this process.

There are at least two possible ways the response of M/T cells can be dissociated: lateral inhibition and back projection modulation. Recent work has shown that back projections can decorrelate mitral cell odor responses, but less in the case of tufted cells^6^.

Lateral inhibition is a prominent motif in many sensory systems, in which neurons receive inhibition from adjacent cells at the same processing level. In the mammalian OB, M/T cells receive their main excitatory input from one glomerulus via their apical dendrite and can receive inhibitory input from several surrounding interneurons in the glomerular, granular and the external plexiform layers^7–17^. These lateral inhibition circuits can modulate M/T cell odor responses so that each cell type carries a different component of the odor stimulus that reflects the response of its main glomerulus and the surrounding glomeruli that also responded to the odor. To better understand how this takes place, the frequency, strength and spatial distribution of lateral inhibition and the way it differentially affects different cell types need to be determined. Despite extensive research in this field, these parameters are not fully known. For example, it is currently unknown how many surrounding glomeruli each M/T cell receives inhibitory inputs from, and whether each of these connections is strong enough to change the neuron’s activity. It also remains unclear whether all projection neurons receive lateral inhibition and whether there are differences in the level of lateral inhibition between cell types.

Several studies have attempted to characterize lateral inhibition *in-vivo* in anesthetized animals using odor stimulation combined with imaging and electrophysiology techniques. These have led at times to contradictory conclusions regarding strength, frequency and spatial distribution. One study found that M/T cells exhibit a patchy center-surround organization reminiscent of lateral inhibition organization in the retina in which inhibitory glomeruli tend to cluster together close to the excitatory glomerulus^18^. By contrast, more recent studies have reached the opposite conclusion that M/T cells receive inhibitory inputs from neurons randomly scattered or semi-organized over the OB surface^17,19,20^. However, odor typically excites several glomeruli and may also directly inhibit some glomeruli due to receptor-odorant interactions or local circuits; hence, using odors to elucidate the inhibitory contribution of each glomerulus and their spatial organization is an indirect form of assessment. *In-vitro* studies typically cannot examine all spatial connections^5,21^. Thus, to date there is no direct estimation of the amount, frequency and spatial extent of lateral inhibition in the olfactory bulb *in-vivo*.

Here we used precise optogenetic stimulations in a transgenic mouse strain to systematically characterize the frequency, strength and spatial distribution of lateral inhibition in different cell types in the mouse OB. Unlike a previous optogenetic approach that used mice expressing Channelrhodopsin2 (ChR2) in M/T cells^22,23^ we used mice that express Channelrhodopsin2 (Chr2) in the olfactory sensory neurons (OSNs) such that optical stimulation resembles odor activation^24^. We found that M/T cells can be divided into at least three functional groups based on the amount and strength of lateral inhibition they receive. These results support a model in which there are several classes of output neurons, each of which receives different levels of surround inhibition, which provides a mechanism for sending different odor features to the cortex in several parallel pathways.

## Materials and Methods

All surgical and experimental procedures were conducted in accordance with the National Institutes of Health Guide for the Care and Use of Laboratory Animals and the Bar Ilan University guidelines for the use and care of laboratory animals in research, and were approved and supervised by the Institutional Animal Care and Use Committee (IACUC).

### Animal preparation

Twenty-three transgenic male and female mice aged 3 to 6 months were used. The animals were housed in a group cage and received no experimental treatment except genotyping. They were maintained in a reverse light/dark cycle and all experiments were performed during their dark cycle.

We used the Omp-ChR2 transgenic mouse line, in which Channelrhodopsin is expressed exclusively in OSNs^24^.

Animals were first anesthetized with ketamine/medetomidine (60/0.5 mg/kg, I.P.) and then fixed in a stereotaxic frame. The bone overlying the dorsal OB was removed. Additional anesthesia was administered as needed (~30% of original dose of ketamine/medetomidine). The animal’s body temperature was maintained at 37 °C using a homeothermic blanket system (Harvard Apparatus).

Ketamine is a known antagonist of NMDA receptors, which might hinder inhibition by granule cells. It is therefore possible that the result here reflects inhibitory circuits that are less affected by ketamine (e.g. glomerular and EPL layer inhibitions, but see also the discussion section).

### Electrophysiology

The spiking activity of neurons was recorded extracellularly using tungsten electrodes (~10 MΩ, FHC, UEWLGESELNNM). Neural signals were amplified and filtered at 300–5,000 Hz (AM-Systems 1800), digitized at 40 kHz (National Instrument, Austin, TX) and stored on a computer. Spike signals were sorted offline using MClust software in MATLAB (written by A.D. Redish).

M/T cells are the sole projection neurons in the olfactory bulb (OB) and are located at a depth of ~200–400 µm from the OB surface^25^. M/T cells are the largest and most abundant neurons at these depths and therefore, as in previous studies, blind recordings at 200–400 µm will mostly result in recordings of M/T cells^9,13,26–32^ as we also recently demonstarted^26^. It is unlikely that we recorded granule cells as previous studies have noted that granule cells are not visible to extracellular electrodes^33–35^. To further verify that we were not recording granule cells, we lowered the electrode to the granule cell layer (~500 µm) and found that no neural activity could be recorded. Furthermore, there was no response to strong light stimulation when the electrode was in the granule cell layer. That said, we cannot completely overrule the possibility that some recorded neurons are not M/T cells.

The electrode was lowered with a micromanipulator (MO-10, Narishige) perpendicular to the OB surface. Neurons were recorded from the dorsal bulb at a depth of ~200–400 µm.

### Optical stimulation of the olfactory bulb

Optical stimulation of the OB has been described in detail in^36^. In brief, M/T cell spike activity was recorded extracellularly in anesthetized mice while optogenetically activating the surrounding glomeruli in the exposed dorsal surface of the OB. Precise spatial control of optical stimulation was achieved by projecting two-dimensional light patterns onto the dorsal surface of the OB using digital micromirror technology (duration 100 ms) coupled to an optical imaging system. This resulted in an image where each projected pixel corresponded to ~11 µm. Optical stimulation was controlled with the MATLAB psychophysical toolbox.

To avoid possible bias due to respiration phase^37,38^, optical stimulation was randomly applied with respect to respiration cycles, with typically 20 repetitions of each spot stimulation. On average 98.34 ± 52.99 spots were stimulated for each recorded neuron.

The light intensity was set to obtain excitatory responses^39^ and ranged from 10–20 mW/mm^2^ as measured by a photometer (Thorlab PM100D). The expression of Chr2 in the OMP-Chr2 mice varies which result in different light power required to elicit a clear response near the recording electrode. Spot sizes ranged from 55 to150 µm (111 ± 27.3 µm, mean ± s.d.). We scanned the full extent of the exposed dorsal OB divided into an N by M non-overlapping spots. N and M were determined by the size of the craniotomy. The result did not change when we only used experiments with the same spot sizes (e.g. 110 µm).

Finally, due to the spatial organization of the axons of passage (i.e., from rostral to caudal)^40^ activation of axons of passage is more common when stimulating the anterior parts of the OB. To avoid this, in a few neurons in which there was a clear response from the anterior OB parts we tended to focus our light stimulation on the more posterior parts of the OB and ignored the anterior parts.

### Data analysis

**Figure 1:**
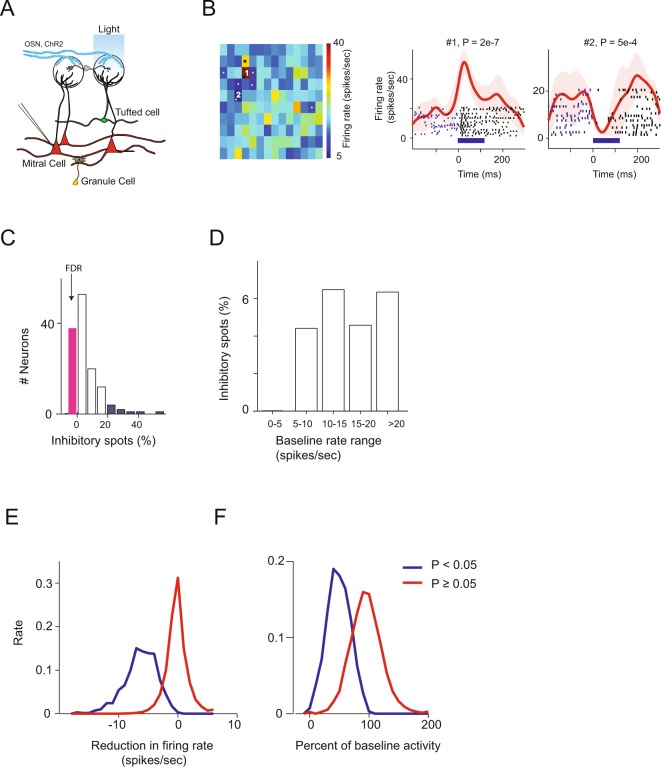
(**A**) Strong lateral inhibition is scarce. Schematic of the experiment for optogenetic stimulation of Omp-ChR2 based mice. ChR2 is expressed in olfactory sensory neurons (OSNs). Light stimulations were randomly applied by scanning the whole exposed OB surface while we recorded from M/T cells. (**B**) Two-dimensional light activation map of a recorded M/T neuron. Each pixel represents the average firing rate in a window of 200 ms from the light onset of each light activated spot, averaged over ~20 randomly interleaved repetitions. Spots that caused a significant change in firing rate are marked with a dot (P < 0.05, Mann-Whitney U-test). Spot size, 111 µm. Right: PETHs and raster plots of one excitatory and one inhibitory spot (marked with numbers). The P values of these excitatory and inhibitory spots are marked (Mann-Whitney U-test). Light stimulations were randomized on the respiration cycle. (**C**) Distribution of the percentage of significant inhibitory spots. The pink bar represents the number of neurons in which the percentage of significant inhibitory spots was lower than the number expected by the false discovery rate (FDR = 3.0% estimated by shuffling the data, Methods). The blue bars represent the neurons that had more than two standard deviations (>20%) of significant inhibitory spots (n = 132). (**D**) Percentage of inhibitory spots as a function of the neurons’ baseline activity. (**E**) The reduction in baseline activity firing rate by the significant inhibitory spots (blue) and the non-significant inhibitory spots (red). (**F**) Same as E but for the percent of change from baseline activity. Only non-excitatory spots were considered.

Data from all animals tested were included in the analysis. Spots were considered inhibitory or excitatory if the mean firing rate across all trials was significantly (P < 0.05) higher or lower than the mean baseline activity 200 ms before light onset. We termed spots that caused a significant reduction in firing rate as strong inhibitory spots. Although statistical significant does not necessarily imply large normalized effect size because statistical significant depends also on the sample size and variance, since in our experiments all spots were stimulated for a similar number of times the P values obtained are comparable to each other and can be used to assess the normalized effect size. Therefore a significant inhibitory spot is likely to have relatively stronger inhibitory effect than a non-significant inhibitory spot (see also text and Fig. 1E,F).

Since we typically light- excited many spots (On average 98.34 ± 52.99 spots) we were likely to have several false positive detections. To estimate the number of false positive detected spots we repeated the same analysis but shuffled the spike count before and after light stimulation of each spot for all trials and recomputed the heat map and the rate of spots that evoked a significant change in firing rate. This shuffling procedure assumes that light stimulation has no effect on the evoked firing rate.

The rate of significant lateral inhibition spots reported in this study is slightly lower than what we reported in^36^. This is because we used a smaller spot size in the range of the size of a glomerulus and scanned the whole dorsal OB in the current study. Peri-event time histograms (PETHs) were smoothed using a Gaussian filter (s.d.: 20 ms).

### Respiration and phase analysis

We recorded respiration using a thermocouple. Because the transition between inhalation and exhalation was the most salient feature in the respiration recording trace, we defined a respiration cycle as being from the beginning of exhalation to the beginning of the next exhalation. The beginning of respiration was thus the same as in^2^. To calibrate our respiration measurements with those conducted in^2^ we compared the respiration signals obtained by a thermocouple, a piezoelectric sensor (APS4812B-LW100-R, PUI Audio, Inc.) and a pressure sensor (1 INCH-Dx-4V-MINI). The piezoelectric and pressure sensor signals were very similar, with 5.9 ± 2.1 ms jitter (mean ± s.d). The thermocouple signal, however, was ~26.3 ± 8.6 ms before the pressure sensor signal. We therefore shifted our respiration estimates by 26.3 ms to be consistent with the beginning of respiration as defined in^2^.

To compute the neurons’ preferred phase we aligned all respirations to the beginning of exhalation and constructed a PETH. The time of the PETH peak relative to the mean respiration duration was computed and used as the neuron’s preferred phase. We considered a neuron to be phase-locked only if the spike time distribution was significantly different from a uniform distribution (Rayleigh test, P < 0.01) and if the peak firing rate was higher from the minimum by at least three spikes/sec. These thresholds detected all phase-locked neurons. Changing the threshold parameters (e.g. setting P < 0.05 or not setting a three spikes/sec difference limit) did not change the results.

Not all recorded neurons were locked to the respiration phase. However, the result of this study did not change if we restricted our analysis to only neurons that were significantly locked to respiration and we therefore did not exclude neurons from our analysis when the neuron preferred phase was not relevant to the analysis.

## Results

### Strong lateral inhibition is scarce

We first characterized the lateral inhibition input of M/T cells in a relatively systematic and comprehensive way. We extracellularly recorded the spiking activity of M/T neurons in the OB while sequentially optically stimulating all exposed glomeruli (~98 spots on average, Fig. 1A,B). We light-stimulated the OB in Omp-ChR2 mice to mimic odor activation, since these mice express ChR2 in the OSNs. Each recorded neuron was typically excited strongly by one-two spots adjacent to the recording electrode and a few spots weakly around it. The magnitude of the light-evoked response was comparable to that observed during odor stimulation (18.3 ± 10.7 spikes/sec)^27,41^. About 3.6% of the light stimulated spots that were not adjacent to the recording electrode also showed significant excitation. This however was expected from estimates of the false discovery rate (3.3%, shuffling analysis, see Methods) which indicated that activation via axons of passage was infrequent in our setup (see also Discussion).

To assess the inhibitory inputs each recorded neuron received from its surround, we first counted the number of light-activated spots that significantly reduced the firing rate of the recorded neuron from its baseline firing rate (P < 0.05, ~20 repeats, Mann-Whitney U test). We found that contrary to what might be expected, inhibitory responses were quite rare. First, in ~29% (38/132) of the recorded neurons none of the light-stimulated spots elicited a significant reduction in baseline firing rate. Second, on average, only 5.3% ± 8.6% (mean ± s.d.) of the surrounding light-stimulated spots elicited a significant inhibition (median 2.5%, range: 0–51%, n = 132 neurons, corrected for the estimated false discovery rate, Fig. 1C, Methods). This estimate is unlikely to be an underestimate since we used spot sizes and a light intensity that elicited clear excitatory responses that should have driven the inhibitory glomeruli. Furthermore, the rate of the inhibitory spots was similar in neurons with baseline firing rates above 5 spikes/sec (Fig. 1D) and there was no correlation between the baseline firing rate and the percentage of inhibitory spots (r = 0.11, P = 0.18, Pearson correlation).

To estimate the strength of these significant inhibitory spots we plotted the absolute and relative reduction in baseline firing rate of all the significant inhibitory spots and the non-significant inhibitory spots (Fig. 1E,F). The mean reduction in baseline activity caused by the significant inhibitory spots was 6.27 spikes/sec (median = 6.14 spikes/sec) compared to an average of 0.17 spikes/sec of the none-inhibitory spots. The mean relative reduction was 51.3% (median = 51.2%) compared to an average reduction of 4.6% of the none-significant spots. Only 6% of the significant inhibitory spots caused a reduction in baseline activity that is lower than 2 spikes/sec and only 6% of the non-significant inhibitory spots caused a reduction in baseline activity that is higher than 3 spikes/sec. This analysis further suggests that the number of strong inhibitory spots is low.

Hence, while this analysis cannot exclude the possibility that there is a group of neurons with low baseline activity that receives strong lateral inhibition, the majority of the recorded neurons received strong lateral inhibition from only a small set of surrounding glomeruli and there was a substantial number of neurons that did not receive strong inhibition from any of the surrounding glomeruli in anesthetized mice.

### Weak lateral inhibition is more common

The above analysis shows that strong lateral inhibition from surrounding glomeruli is infrequent. Many neurons do not receive strong lateral inhibition at all. Those that do, experience inhibition from a handful of surrounding glomeruli. However, our estimation of the percentage of surrounding inhibitory glomeruli was based on a threshold test. In other words, a spot was considered inhibitory if its light activation caused a significant reduction in the firing rate of the recorded neuron (P < 0.05). However, some of the light-activated spots evoked weak inhibition that might not have crossed this sharp and somewhat arbitrary threshold (P = 0.05). Thus, this procedure is biased towards detecting spots that exert a relatively strong inhibition and is more likely to fail when there is weak inhibition.

Examining more carefully the mean reduction in baseline activity caused by the none-significant inhibitory spots (Figs. 1E,F, red lines) we noticed that while the average and median values were very close to zero (mean = −0.17, median = −0.2 spikes/sec) they were significantly different from zero (P < 1e-39 for the mean and median, one sample t-test and sign test respectively). This strongly suggests that our classification method of inhibition does not capture all inhibitory spots and it is biased towards strong ones.

**Figure 2:**
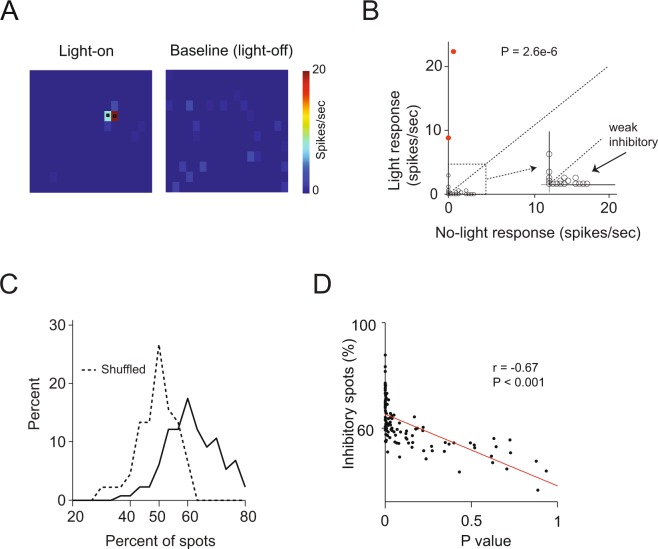
Weak lateral inhibition is more common. (**A**) Two-dimensional light activation map of an M/T neuron. Left panel shows the light-evoked mean response and the right panel shows the baseline activity 200 ms before light stimulation. Spot size 88 µm. (**B**) An identity graph showing the mean firing rate before and after light onset of all spots in the example shown in A. Most light stimulated spots caused a small insignificant reduction in the firing rate. Considering all the non-excitatory spots, the firing rate of the neurons before light onset was significantly higher than when the light was on (P = 2.6e-6, Mann-Whitney U test). Inset shows a zoom-in of the dots near the axes intersection. Red filled dots are the two spots classified as excitatory. (**C**) Distribution of the percentage of light-activated spots that caused a reduction in the baseline activity. The percentage of spots that caused any reduction in the baseline activity could reach 80% and was higher than 62% in half of the neurons. The dashed line shows the distributions when we shuffled the data which was centered on 50% as expected. (**D**) Correlation between the percentage of spots that reduced the baseline activity in each neuron and the P value for each recorded neuron (r = −0.66, P < 0.001, Pearson correlation).

To estimate the extent of weak inhibitory inputs we devised a new method in which we compared the mean response before and after light stimulation of all none excitatory spots (paired t-test, P < 0.05). This method provides a stronger statistical power as it pools all average spot responses and therefore can capture small reductions in baseline activity as long as it is caused by a substantial number of spots. To see this, consider the response map example shown in Fig. 2A. In this example, none of the light- activated spots crossed the threshold to be considered inhibitory per se. However, numerous spots that were not classified as inhibitory evoked a response that reduced the activity of the recorded neuron below baseline. In fact, when we compared the mean firing rate evoked by light activation of all these presumably ‘non-inhibitory’ spots to the mean baseline activity, we found that there was a small but highly significant reduction in firing rate (P = 2.6e-6, paired t-test, n = 216 spots, spot size 88 × 88 mµ, each spot value is the represented by the average of its ~20 repetitions, Fig. 2B). This suggests that in addition to the strong but sparse inhibitory connections reported previously (Fig. 1), there were also weak inhibitory inputs from presumably several surrounding glomeruli. To quantify this weak surround inhibition we conducted this comparison of the mean firing rate before and after light stimulation of all the non-excitatory spots to all recorded neurons. When applying this criterion with P < 0.05 as a threshold, we found that 40% (15/38) of the neurons that did not receive significant inhibition from any of the light-stimulated spots did receive significant weak inhibition.

To estimate the number of surrounding glomeruli that elicited inhibition (weak or strong) we computed the percentage of spots that caused any reduction in baseline firing rate. We found that the distribution of the percentage of spots that caused any reduction in baseline activity was normally distributed around 62% and could reach up to 80% in a few M/T cells (Fig. 2C). The percentage of spots that caused a reduction in baseline activity when we shuffled the spikes count before and after light stimulations was 50%, as expected. These two distributions were significantly different (P = 1.4e-11, Kolmogorov Smirnov test). This analysis suggests that on average ~12% of the surrounding spots caused some reduction in baseline activity. This rate of inhibitory connections is twofold higher than the rate of significant inhibitory connections (e.g. 5.3% Fig. 1C,D). Note that the P value of this statistical test was highly correlated to the percentage of spots that caused reductions in baseline activity (r = −0.66, P < 0.001, Fig. 2D). This suggests that this measure of inhibition quantifies the total number of (weak and strong) inhibitory inputs.

### Lateral inhibition depends on the recorded neuron type

While on average significant lateral inhibition was not common there was substantial variability in the number of spots that elicited significant inhibition. Specifically, a large group of recorded neurons did not receive significant inhibition from any of the surrounding glomeruli whereas a small group received inhibition from as much as 20–51% of the surrounding glomeruli (Fig. 1C). Five neurons received inhibition from more than 25% of the surrounding spots and eight from more than 20% of the spots (Fig. 1C). We next examined whether this variability was related to the type of recorded neuron.

**Figure 3:**
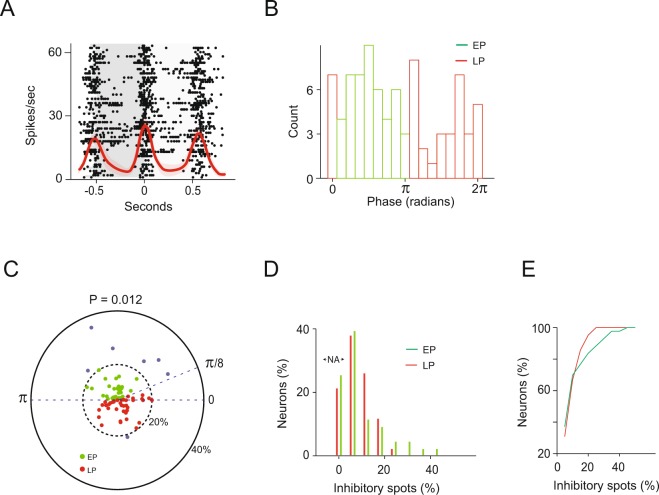
The percentage of strong lateral inhibition depends on the recorded neuron type. (**A**) An example of phase locking of an M/T cell. The gray shadings mark the different respiration cycles. Zero is defined as the transition between inhalation and exhalation. This neuron was locked to the transition between exhalation and inhalation. Peri-event time histograms (PETHs, mean ± s.e.m.) and raster plots are shown. (**B**) The distribution of all neurons’ preferred phases. Only neurons with a significant preferred phase are plotted (N = 85 neurons, methods). EP cells are marked in green and LP cells in red. Red and green bars show the estimated boundary between EP and LP cell phases. Two boundaries for EP were examined: 0 to π or π /8 to π. The latter boundary was suggested by^2^ as separating tufted from mitral cells. (**C**) A polar plot of the percentage of inhibitory spots as a function of the neurons’ preferred phase. Dashed lines show the estimated boundary between EP and LP cell phases (see **B**). Green dots mark EP cells and red dots mark LP cells. The blue dots mark the six EP neurons and one LP cell in which the percentage of inhibitory spots exceeded 20%. The percentage of inhibitory spots were more variable for EP (P = 0.023, Levene’s test when using the 0 to π boundary and P = 0.012 when using the π/8 to π boundary to define EP cells). (**D**) Percent of neurons as a function of the percent of inhibitory spots. Only EP cells (green) received dense lateral inhibition (e.g. >20%). (**E**) Accumulative distribution of the data shown in D.

A recent study has suggested that M/T cells can be identified unambiguously based on their preferred sniff phase in intra-cellular or extra-cellular recordings^2,42^. Tufted cells seem to fire during the first half of the respiration cycle (π/8 to π radians), while mitral cells tend to fire during the second half of the respiration cycle (π to π/8 radians). Consistent with studies that have examined neurons’ preferred phases in a large number of M/T cells^2,27,29,43^ we also observed that the recorded neurons’ preferred sniff phases were widely distributed across the sniff cycle (Fig. 3A,B).

To evaluate the ways in which lateral inhibition depends on the M/T cell respiration preferred phase we plotted the percent of significant inhibitory spots of each of our recorded neurons as a function of its respiration preferred phase (Fig. 3C, n = 85 neurons, only neurons that were significantly phase locked were used, see Methods).

We first noticed that all neurons receiving significant lateral inhibition from many spots (e.g. five neurons receiving inhibition from more than 25% of the surrounding spots) and almost all neurons receiving relatively dense levels of surround inhibition (6 out of 7 neurons receiving inhibition from more than 20% of the surrounding spots) were phase-locked to a relatively narrow band in the first half of the respiration cycle (e.g., early phase neurons, EP, Fig. 3C). Note that the probability that all five neurons would fall in the first half of the respiration cycle is 0.03, and the probability that 6 out of 7 cells would fall in the first half of the respiration cycle is 0.008 (binomial tests). Overall, there was a significant difference in the variability of the percent of significant inhibitory spots between EP and LP cells, with the EP cells exhibiting a much larger variance (P = 0.012, two-sample Levene’s test, F = 6.5, df_1_ = 1 df_2_ = 83).

The finding that EP and LP neurons differ in their level of lateral inhibition may reflect differences in the level of lateral inhibition of mitral and tufted cells. However, since we could not directly verify the neuron types in our experimental setup, in what follows they are only referred to as EP and LP cells. We note that all the results reported in this study were still statistically significant when we defined EP as starting at zero and not at π/8 radians (and LP as ending at 2π). This may imply that the differences we found are not between mitral and tufted cells but rather between early and late phase spiking cells. The differences between these two neuron types cannot be explained by differences in their baseline firing rates because the EP and LP baseline firing rates were similar (P = 0.49, two tailed two sample t-test, t = 83).

These findings suggest that the percentage of surrounding glomeruli eliciting strong lateral inhibition is more variable in EP cells. A subset of EP cells receives inhibition from a large number of surrounding glomeruli whereas LP neurons tend to receive strong inhibition from only a relatively small number of surrounding glomeruli (Fig. 3D,E).

### Weak lateral inhibition is relatively more common in LP than in EP cells

**Figure 4:**
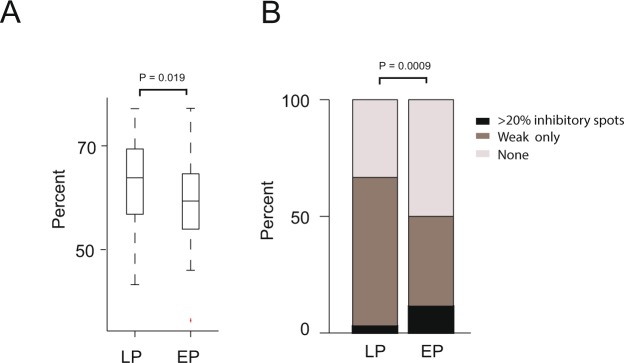
Weak lateral inhibition is relatively more common in LP than in EP cells. (**A**) A box plot showing the percentage of spots that caused any reduction in baseline activity in LP and EP neurons. LP cells tended to have more spots that reduced the baseline activity compared to EP cells (P = 0.019, two-tailed t-test). (**B**) A breakdown of the different types of inhibition in EP and LP cells into three major groups: (1) Percent of cells which had at least 20% significant inhibitory spots (black); (2) Weak: percent of cells with significant overall reduction in baseline activity following light stimulations across all stimulated spots; (3) None: percent of neurons which received no significant inhibition at all (weak or strong).

We next examined whether EP and LP cells differed in their level of weak inhibition. To address this, we focused on neurons that have a small number of significant inhibitory spots (e.g. less than 20%, similar results were obtained when we focused on less than 10% or 15%). This allowed us to examine neurons which mostly receive weak inhibition or no inhibition. Comparing these neurons’ percent of spots that caused any reduction in baseline activity and their preferred phase showed that LP cells tended to have more spots that caused a reduction in baseline activity (P = 0.019, Mann Whitney U-test, Fig. 4A). In other words, the LP neurons which tend not to receive strong inhibition receive more weak inhibition compared to EP cells. Together with the previous analysis (e.g. Fig. 3C,D), these results show that EP and LP cells differ in the levels of lateral inhibition: LP cells tend not to receive strong lateral inhibition from many surrounding glomeruli but they receive more weak inhibition while some EP cells receive strong lateral inhibition from a large number of surrounding glomeruli but receive less weak inhibition. Fig. 4B summarizes the differences in lateral inhibition of EP and LP. The difference between these two cell groups was highly significant (P = 0.0009, Fisher exact test).

### Proximal neurons can receive different levels of inhibition

**Figure 5:**
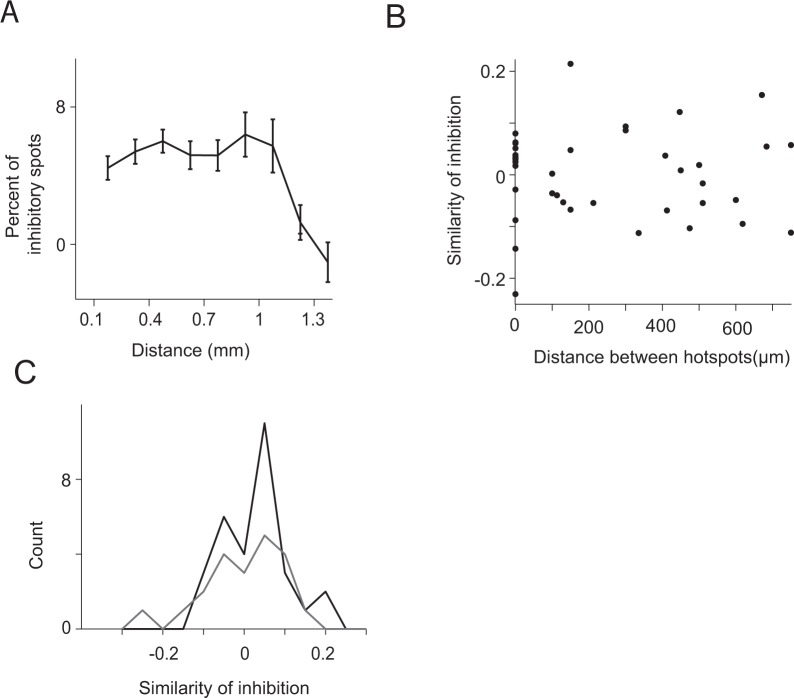
Proximal neurons can receive different levels of inhibition. (**A**) The mean percentage of significant inhibitory spots (FDR substructed) as a function of the distance from the main excitatory spot. The percentage of inhibition was similar up to 1 mm and then dropped to zero. The slightly lower rates of inhibitory spots near the excitatory spots are probably an artifact caused when the light stimulation was close to the recorded neuron, which can sometimes slightly increase the recorded neuron’s firing rate and thus reduce the probability of detecting the inhibition. (**B**) A scatter plot of the differences in inhibition levels of all pair-wise neurons recorded in the same mouse. The same neuron could participate in more than one pair. The distance between two neurons was estimated by computing the distance between the two neurons’ main hotspot. The similarity in inhibition levels between two neurons was estimated by computing the difference between the percent of spots that caused any reduction baseline activity. No correlation was detected. (**C**) The distribution of inhibitory input similarity for neurons recorded on the same electrode (gray) and these recorded at different depths or locations (black). Neurons recorded on the same electrode could receive different levels of inhibition.

We next examined the spatial distribution of lateral inhibition. A comparison of the rate of significant inhibitory spots as a function of the distance from the recorded neuron showed that the rate of inhibitory spots was relatively constant up to 1 mm from the recording neuron and diminished more distally (Fig. 5A). To examine the effect of more distant glomeruli we repeated the experiment while recording M/T cells located in the ventral parts of the OB and light stimulated the dorsal OB. There was no significant inhibition from any of the light-stimulated spots (N = 15 neurons) in any of the recordings. Examining the locations of the significant inhibitory spots we found that they were spread randomly over the bulb surface.

We next inquired whether the amount of inhibition any two neurons receive from their surround depends on their physical location in the OB by comparing the similarity in inhibition to the distance between the neurons’ primary excitatory spot. The distance between the primary excitatory spots is known to be a good approximation of the distance between two neurons^44^. Similarity in the levels of inhibition was quantified by comparing the percent of significant inhibitory spots of each neurons (i.e. strong inhibition) or comparing the percent of spots that caused any reduction in baseline activity (i.e. weak and strong inhibition). We found that inhibition similarity was independent of the distance between the neurons as even proximal neurons could receive dissimilar amounts of inhibitory inputs (P = 0.19, and P = 0.96 for two methods used to define similarity in lateral inhibition, Pearson correlation, Fig. 5B). This absence of correlation persisted when we tested EP and LP cells separately (P > 0.2 for both LP and EP cells). Considering only neurons recorded on the same electrode revealed they also differed in the level of inhibition they receive (n = 25 neuron pairs) as non-simultaneously recorded neurons (Fig. 5C). These analyses suggest that lateral inhibition sources are randomized over the bulb surface and that even neurons that are located close to each other can receive different levels of inhibition. Thus, M/T cells that receive similar odor inputs appear to be modulated by different inhibitory circuits that provide a mechanism for sending different odor features to the cortex in several parallel pathways.

## Discussion

Although many previous studies investigated lateral inhibition in the OB there is a lack of systematic characterization and quantification of basic parameters. In this study we characterized the frequency, strength and spatial organization of lateral inhibition that M/T cells receive from all exposed dorsal glomeruli in the OB. We found that strong lateral inhibition is rare and was seen in only ~5–6% on average of the surround glomeruli. This sparsity of strong inhibitory connectivity is consistent with a previous estimation in rats, which predicted 2%^19^.

While overall strong inhibition was quite un-common a different picture emerged when the lateral inhibition of EP and LP cells was considered separately and when we distinguish between strong and weak inhibition. We found that LP cells primarily received weak inhibition whereas EP cells received strong, weak or no inhibition at all (Fig. 4B). We therefore identified three different groups of neurons that differed in their level of lateral inhibition. (1) Neurons receiving strong inhibition (mostly EP cells) (2) Neurons receiving mostly weak inhibition (both EP and LP cells but more common in LP cells) and (3) Neurons that do not receive any detectable inhibition (both EP and LP cells but more common in EP cells).

We note that our definition of strong inhibition relay on statistical significance test (e.g. P < 0.05) which might miss few strong inhibitory changes that are highly variable or include weak inhibitory changes that have low variance. Therefore one should interpret ‘strong’ as relating to the normalized effect size (because all neurons were stimulated with a similar number of repetitions per spot). Furthermore, Fig. 1E,F show that our P-value criterion mostly captures inhibitory spots that caused a substantial absolute change in firing rate.

The existence of several types of lateral inhibition circuits suggests that lateral inhibition plays several roles in addition to decorrelating odor responses. One possible role of the subgroup of neurons that do not receive lateral inhibition could be to detect the existence of an odor. Quick detection at a low odor concentration is crucial because it can direct the animal towards (or away) from an odor source and can be used to immediately change sniffing strategy^45^ and/or activate cortical processing. Thus, the group of neurons that do not receive any weak or strong lateral inhibition can signal the brain that an odor has been detected without any attenuation or delays in their firing. Once the presence of an odor is detected, decorrelating the responses can improve the animal’s ability to identify and discriminate odors, as was shown in^6,14,46^. This may be the role of the EP cells and some LP cell that receive strong lateral inhibition. Finally, the group of LP cells that receive weak lateral inhibition from many surrounding glomeruli could act to maintain responses across several odor concentrations. Increasing odor concentration typically increases the neuron’s firing rate but also increases the response of the surrounding glomeruli. When there are many inhibitory connections, the increase in response from the increase in odor concentration can be canceled out or attenuated by inhibition from the surrounding glomeruli, thus resulting in a similar response to several odor concentrations.

Our results are based on optogenetic stimulations. Although optogenetics is well suited to revealing the contribution of specific glomerulus compared to odor or electrical stimulation, it has several limitations. Similar to electrical stimulation, optogenetic stimulation of the OSNs may activate axons of passage; hence, activating one glomerulus can cause the activation of other more posterior glomeruli. While this may have occurred here, it is unlikely to have significantly biased our results. This is because only very few spots outside the primary spatial zone elicited a statistically significant excitation in M/T cells (3.6%, Mann-Whitney U-test, P < 0.05, estimated false discovery rate: 3.3%), indicating minimal fiber stimulation. This is consistent with a previous study also reporting that the threshold for activating M/T cells was much lower in glomeruli than in axons of passage^24^. To estimate the possible contribution of activation of axons of passage to our analysis, we compared the reduction in spike count caused by all the stimulated spots anterior to the excitatory spots to those posterior to the excitatory spots. The reduction in firing rate caused by stimulating the posterior spots did not differ significantly from the reduction caused by stimulating the anterior spots (P = 0.13, t-test). All this suggests that under the experimental conditions reported here, the effect of activating axons of passage on neural activity was negligible.

Another limitation of light (and electrical) stimulation is that light may only reach parts of a glomerulus or can hit several neighboring glomeruli. This can result in insufficient activation or activation of several glomeruli. While possible, we consider that this problem did not skew our results substantially due to the relatively large number of surrounding spots tested (98.34 ± 52.99 spots per neuron, mean ± s.d.) which should have averaged out instances of activation of too many or too few glomeruli.

We found that lateral inhibition is spread over the OB surface up to ~1 mm away from the recorded neuron. This is consistent with previous studies which reported that inhibition and excitation were intermingled, and runs counter the center-surround model at least in its classical definition as in the retina^18–20^. We also found that proximal neurons could receive completely different levels of inhibition (Fig. 5B). This is consistent with the view that the glomerulus organization on the OB surface is only coarsely organized by odor tuning similarity^47^.

Finally, our experiments were conducted in ketamine-anesthetized animals. Ketamine is a known antagonist of NMDA receptors, which might hinder inhibition by granule cells^48^. It is therefore possible that the result here reflects inhibitory circuits that are less affected by ketamine (e.g. glomerular and EPL layer inhibitions). However, it is worth noting that previous examination of lateral inhibition under urethane anesthesia also reported low rates of lateral inhibition^19,22^. This might suggest that the effect of granule cells on M/T cell baseline firing rates is not very strong. Consistent with these results, recent studies reported that silencing granule cells did not substantially affect M/T cells activity^42,49^. However, direct examination of granule cells effect on M/T cells in a wake mice is required to better understand their role in lateral inhibition and other odor processing tasks.

Thus overall, despite extensive research and seminal discoveries, the mechanisms by which odors are encoded in the OB are still elusive. Our results show that lateral inhibition can differentially shape the responses of the different OB projections neurons into at least three possible parallel streams. These results have important implications for the ways in which odors are encoded in the OB, how different odor features are transmitted in parallel to the cortex, and how the cortex processes odor information.
